# *In Vitro* and *Ex Vivo* Testing of Tenofovir Shows It Is Effective As an HIV-1 Microbicide

**DOI:** 10.1371/journal.pone.0009310

**Published:** 2010-02-19

**Authors:** Lisa C. Rohan, Bernard J. Moncla, Ratiya Pamela Kunjara Na Ayudhya, Marilyn Cost, Yunda Huang, Fang Gai, Nicole Billitto, J. D. Lynam, Kara Pryke, Phillip Graebing, Nicole Hopkins, James F. Rooney, David Friend, Charlene S. Dezzutti

**Affiliations:** 1 Magee-Womens Research Institute, University of Pittsburgh, Pittsburgh, Pennsylvania, United States of America; 2 University of Pittsburgh, Pittsburgh, Pennsylvania, United States of America; 3 Fred Hutchinson Cancer Research Center, Seattle, Washington, United States of America; 4 Gilead Sciences, Inc., Foster City, California, United States of America; 5 CONRAD, Arlington, Virginia, United States of America; University of California, San Francisco, United States of America

## Abstract

**Background:**

Tenofovir gel has entered into clinical trials for use as a topical microbicide to prevent HIV-1 infection but has no published data regarding pre-clinical testing using *in vitro* and *ex vivo* models. To validate our findings with on-going clinical trial results, we evaluated topical tenofovir gel for safety and efficacy. We also modeled systemic application of tenofovir for efficacy.

**Methods and Findings:**

Formulation assessment of tenofovir gel included osmolality, viscosity, *in vitro* release, and permeability testing. Safety was evaluated by measuring the effect on the viability of vaginal flora, PBMCs, epithelial cells, and ectocervical and colorectal explant tissues. For efficacy testing, PBMCs were cultured with tenofovir or vehicle control gels and HIV-1 representing subtypes A, B, and C. Additionally, polarized ectocervical and colorectal explant cultures were treated apically with either gel. Tenofovir was added basolaterally to simulate systemic application. All tissues were challenged with HIV-1 applied apically. Infection was assessed by measuring p24 by ELISA on collected supernatants and immunohistochemistry for ectocervical explants. Formulation testing showed the tenofovir and vehicle control gels were >10 times isosmolar. Permeability through ectocervical tissue was variable but in all cases the receptor compartment drug concentration reached levels that inhibit HIV-1 infection *in vitro*. The gels were non-toxic toward vaginal flora, PBMCs, or epithelial cells. A transient reduction in epithelial monolayer integrity and epithelial fracture for ectocervical and colorectal explants was noted and likely due to the hyperosmolar nature of the formulation. Tenofovir gel prevented HIV-1 infection of PBMCs regardless of HIV-1 subtype. Topical and systemic tenofovir were effective at preventing HIV-1 infection of explant cultures.

**Conclusions:**

These studies provide a mechanism for pre-clinical prediction of safety and efficacy of formulated microbicides. Tenofovir was effective against HIV-1 infection in our algorithm. These data support the use of tenofovir for pre-exposure prophylaxis.

## Introduction

The recent report from the Joint United Nations Programme on HIV/AIDS (UNAIDS) suggests the HIV-1 epidemic has stabilized [1]. Despite this encouraging news, this past year, 2.7 million new infections and 2 million more people have perished as a consequence of HIV-1 infection. A renewed effort in the HIV-1 prevention field has taken place over the past decade. As the majority of new infections occur through sexual intercourse, new approaches in addition to vaccine development have been pursued and include male circumcision, treatment of sexually transmitted infections, and pre-exposure prophylaxis (PrEP). All have had varying degrees of success [2], [3], [4], [5].

Sub-Saharan Africa encompasses the majority of the global HIV-1 infected population [1]. Women now account for approximately half of the infected overall population and greater than 60% in sub-Saharan Africa. A major gap in prevention strategies is the empowerment of the receptive sexual partner with the means to prevent HIV-1 acquisition that do not depend on their partners' consent. This is especially important where the receptive partner may have difficulty negotiating the use of condoms [6], [7], [8]. One of the most promising concepts is microbicides which are products used topically, either vaginally or rectally, or taken orally to prevent the transmission of HIV-1. Microbicides are a form of PrEP and have the potential to be used without the partner's knowledge.

While there are several broad classes of microbicides, three major ones are described. One class of microbicides bolsters the natural physiological conditions of the vagina by providing a pH buffer (e.g. BufferGel and ACIDFORM) [9], [10]. A low pH like the vaginal environment inactivates both HIV-1 and HSV-2; the neutral pH of the rectum may limit the utility of this product. A clinical efficacy study was just completed and the results showed that BufferGel was quite safe and acceptable, but not effective at preventing HIV-1 acquisition [11]. A second class of microbicides blocks the fusion of HIV-1 with its target cells and is a large group with diverse structures; examples include cellulose sulfate, Carraguard, and PRO 2000. All three of these products have been evaluated in efficacy trials where the primary endpoint was HIV-1 infection. The cellulose sulfate trials were halted early for futility or a suggestion of harm [12], [13]. The Carraguard trial demonstrated the product was safe and acceptable by the women using it, but not effective at preventing HIV-1 infection [14]. Of the two trials evaluating PRO 2000, the MDP301 trial had one of the two testing arms stopped due to futility (2% PRO 2000) while the other arm (0.5% PRO 2000) completed testing. The HPTN 035 trial demonstrated that 0.5% PRO 2000 was 30% effective in preventing HIV-1 infection [11]. The 0.5% PRO 2000 result was not statistically significant and was confirmed by the recently completed MDP301 trial [15].

A third class of microbicides interferes with the life cycle of HIV-1 and includes drugs that are currently used for treatment (e.g. tenofovir) or have been evaluated for treatment (e.g. UC781 and Dapivirine). Tenofovir, a nucleotide analog HIV-1 reverse transcriptase inhibitor, is the most extensively studied. Currently, tenofovir is given orally in the form of the prodrug tenofovir disoproxil fumarate (TDF) which is effective against a range of HIV-1 subtypes as well as CCR5-using and CXCR4-using HIV-1 and is being proposed for use in PrEP clinical trials [16]. In addition to the oral dosage form, a vaginal gel product has been developed which contains 1% of the non-prodrug form, tenofovir. The formulated topical gel (tenofovir gel) has been tested in non-human primate models for pre- and post-exposure prophylaxis with varying degrees of success [17], [18] and two human safety trials with favorable results [19], [20].

**Figure 1:**
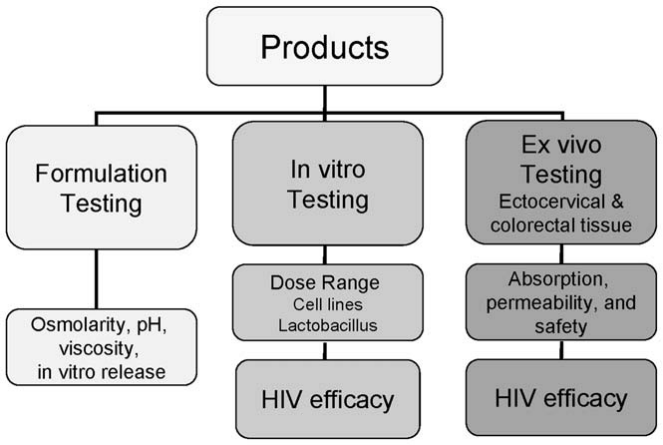
Microbicide pre-clinical testing algorithm. Tenofovir and vehicle control gels were evaluated through a comprehensive pre-clinical algorithm. The algorithm focuses on testing the formulation's physiochemical properties and the ability to release the drug; *in vitro* testing that includes safety of vaginal flora, epithelial and immune cells, and efficacy against multiple HIV-1 clades; and *ex vivo* testing using ectocervical and colorectal explants to evaluate drug absorption and formulation safety and efficacy against HIV-1. The data obtained from this algorithm along with data supplied by the manufacturer aid in the decision to continue testing the product.

The overall goal of this work was to evaluate the tenofovir gel formulation for safety and efficacy using our pre-clinical testing algorithm. We have a comprehensive approach for analyzing the physical attributes of the formulation (viscosity, osmolality, *in vitro* release, tissue permeability), testing for safety (genital tract flora, epithelial cells, and ectocervical and colorectal tissue explants), and demonstrating efficacy (prevention of HIV-1 infection in peripheral blood mononuclear cells [PBMCs] and ectocervical and colorectal explants) (Figure 1). The ability to model systemically available drugs, specifically tenofovir, against mucosal HIV-1 infection using *ex vivo* assays has not been previously accomplished. Therefore, our second goal was to determine whether systemically available tenofovir could be modeled *ex vivo* for efficacy. While our data show that the topical tenofovir gel formulation was hyperosmolar which was reflected in changes in epithelial monolayer integrity and explant epithelium fracture, it was safe for normal vaginal flora and effective in the PBMCs and explant cultures against HIV-1 challenge. Moreover, systemic administration of tenofovir was also effective at preventing HIV-1 infection of the ectocervical and colorectal explant cultures. Collectively, these data suggest that tenofovir is an excellent candidate as a topical vaginal or rectal microbicide and for oral PrEP. Our intent is to validate our pre-clinical algorithm with the findings from the on-going tenofovir gel and PrEP efficacy trials. Rigorous evaluation of formulated products prior to inclusion in large efficacy studies should be done to ensure successful outcomes.

## Materials and Methods

### Products

Tenofovir gel also known as 9-[2-(phosphonomethoxy)propyl]adenine (PMPA) gel, vehicle control gel, and tenofovir powder were provided by Gilead Sciences, Inc. (Foster City, CA) and CONRAD (Arlington, VA). Tenofovir gel is composed of 1% tenofovir incorporated into a formulation containing a gelling agent (hydroxyethycellulose), glycerin, EDTA, citric acid, and the preservatives methyl and propyl parabens. The vehicle control gel was the same formulation but without the active ingredient, tenofovir. While tenofovir disoproxil fumarate is the oral prodrug of tenofovir, it was not provided or used for this work. A 10 mg/ml solution of the tenofovir powder was prepared as described below. Where appropriate, Gynol II (Ortho-McNeil-Janssen Pharmaceutical, Inc. Titusville, NJ) an over-the-counter 3% nonoxynol 9 (N9) gel was used as a positive control for cell and tissue toxicity.

### Human Tissue

Normal human ectocervical (IRB # 0503103) and colorectal (IRB # 0602024) tissues were acquired from pre-menopausal women undergoing hysterectomy or persons undergoing colorectal surgery for non-inflammatory conditions, respectively. All tissue was obtained through IRB approved protocols at the University of Pittsburgh. The IRB deemed this exempt because surgical tissue remainders that would otherwise be discarded are used for this research. No patient identifiers are provided and all tissues are collected through an Honest Broker de-linking patient ID to the investigators. When the patients consent for surgery, they sign a general consent that their tissue remainders can be used for research; therefore, a study specific consent form was not deemed necessary.

### Physicochemical Testing

The major physicochemical parameters typically evaluated for semi-solids include viscosity, osmolality, pH, and *in vitro* drug release. Likewise, permeability of the drug through tissue barriers is an important parameter and was evaluated to determine whether the drug has the capacity to reach target cells or become systemically available.

Viscosity was determined using the CP41 spindle on a cone/plate Brookfield Model DVIIIþ viscometer (Brookfield Eng. Lab., Inc., Middleboro, MA). Data was collected using Rheocalc software (Brookfield Eng. Lab., Inc.). The sample was placed in the sample cup of the instrument and allowed to equilibrate to 37°C for 10 min. Viscosity was measured using a program where shear rate was increased from 0.2 to 60.0 sec^−1^ and subsequently decreased to 0.2 sec^−1^. To compare data across samples, viscosity values acquired at 60 sec^−1^ were used in the analysis. Yield stress was calculated by Rheocalc Software using the Bingham equation which gave the best fit.

pH was determined using an Accumet AR20 pH meter (Fisher) with an AccuFet (Fisher) semisolid-state probe calibrated using two points, pH 4.0 and 7.0.

Osmolality was determined using a Vapor Pressure 5520 Osmometer (Wescor, Inc., Logan, UT) calibrated with Opti-mole 290 and 1000 mmol/kg osmolality standards.

*In vitro* release studies were carried out using the Hanson Microette® system. Spectra/Por membrane discs (Spectrum Chemical Mfg. Corp., New Brunswick, NJ) were used as the inert membrane and preconditioned with a 1 mM EDTA (Spectrum Chemical Mfg. Corp.) solution, the receptor medium was phosphate buffered saline. The membrane was placed on the Hanson Microette® system with a premeasured amount of tenofovir gel. Samples were taken from the receptor compartment at predetermined time intervals and assayed for tenofovir content using high pressure liquid chromatography methods.

Permeability studies (drug moving from the gel through tissue barriers) were conducted in a Franz diffusion cell. The Franz cell is a two-compartment system consisting of an upper chamber (donor compartment) and a lower chamber (receiver compartment). The Franz cells were water-jacketed and temperature was maintained at 37°C throughout the experiment via a circulating water bath. DMEM (Dulbecco's Modification of Eagle's Medium, Mediatech, Manassas, VA) was used in the receiver chamber. The receiver chamber was continuously stirred by magnetic stir bar. The volume of the receptor chamber was 4.8 mL. Ectocervical tissue was used within 2 h of surgery and placed in a Franz diffusion cell separating the donor compartment from the receptor compartment. The epithelial side of the tissue was oriented toward the donor compartment. The tissue was placed on the top of a 7 mm Franz cell opening; which provided a diffusion area of 0.385 cm^2^. The tissue was equilibrated with DMEM in the donor compartment for 5 min prior to the permeability study. After the equilibration period, the DMEM solution was removed from the donor compartment and replaced with 450 µL of tenofovir product. Fifty µL was removed from the donor compartment for time zero HPLC quantitation for tenofovir. Samples were obtained from the receiver compartment at predetermined time intervals for a total period of 6 h and fresh medium was replaced to maintain sink conditions. Samples were assayed for tenofovir using high pressure liquid chromatography methods.

### Safety Testing

Safety encompassed normal vaginal flora, epithelial cell lines, PBMCs, and ectocervical and colorectal explant cultures. Viability of the bacteria, cells, and explants was measured after exposure to the tenofovir and vehicle control gels. Additionally, epithelial monolayer integrity as measured by transepithelial resistance (TER) was tested.

#### Normal vaginal flora testing

One reference strain for each species was obtained from the American Type Culture Collection (Manassas, VA.). Field isolates for each species were obtained from human vaginal samples and identified to the species level using Gonchek II® for *N. gonorrhoeae* (PML Microbiologicals) and confirmed using the Strand Displace Assay as previously described [21]. *Lactobacillus* species were identified using colony morphology, Gram-stain reaction and catalase production and confirmed using DNA-DNA hybridization to DNA from reference strains [22]. The species analyzed included *L. crispatus* (8 strains), *L. jensenii* (11 strains), *L. iners* (10 strains), and *L. vaginalis* (10 strains). *L. vaginalis* strains were further classified by sequencing 16s and 23s ribosomal RNA. Organisms were stored at −80°C in litmus milk until needed. Stock cultures were revived by plating onto either blood agar plates (Columbia blood agar base, PML Microbiologicals, Wilsonville, OR) for *Lactobacillus* or Chocolate agar (PML Microbiologicals or prepared in-house) for *Neisseria gonorrhoeae*. *Prevotella bivia* and *P. melaninogenica* were isolated and cultured as described previously [23].

Minimum cidal concentrations, the drug concentration required to reduce the viability of a culture by 99.99%, were determined as previously described [24], [25]. Briefly, bacterial suspensions were prepared by selecting isolated colonies from fresh overnight culture plates and suspending the test organisms in saline to a density of a 0.5 McFarland standard. Suspensions were diluted in sterile saline and then further diluted in 0.5 mM ACES buffer pH 7.0. After incubation at 35°C for 30 min, samples were plated on to the appropriate medium, allowed to absorb for 10 to 15 min, and then spread over the surface of the agar plate. Plates were incubated as described above for 24 h and evaluated for killing of the test microorganisms by examination. Samples yielding 10 or fewer colony forming units (representing a 99.99% kill) were considered sensitive to killing. All results were compared to the control which was identical but lacking tenofovir. Tenofovir powder was suspended in ACES buffer to 10 mg/ml and was filter sterilized. The tenofovir solution was tested as described above.

#### Epithelial cell lines and PBMCs testing

Epithelial cell lines were obtained from the American Type Culture Collection (Manassas, VA). Unless otherwise stated, culture reagents were purchased from Hyclone (Logan, UT). Caco-2 cells, a colorectal epithelial cell line [26], were grown in MEM alpha modified medium supplemented with 20% heat-inactivated fetal bovine serum (FBS; Gemini Bio-products, West Sacramento, CA), 100 µg/ml streptomycin, 100 U/ml penicillin, and 100 mM L-glutamine. HEC-1-A cells, an endometrial epithelial cell line [27], were grown in McCoy's 5A medium supplemented with 10% FBS, 100 µg/ml streptomycin, 100 U/ml penicillin, and 100 mM L-glutamine. Normal human PBMCs (Central Blood Bank, Pittsburgh, PA) were obtained by leukophoresis from HIV-1-negative blood donors, purified by differential centrifugation, and stored in the gas phase of liquid nitrogen until needed. PBMCs were grown in RPMI-1640 (cRPMI) supplemented with 10% FBS, 100 µg/ml streptomycin, 100 U/ml penicillin, and 100 mM L-glutamine. For activation, PBMCs were stimulated for 3 days at 37°C/5% CO_2_ in cRPMI medium supplemented with 10% interleukin-2 (Roche Applied Sciences, Indianapolis, IN) and 5 µg/ml phytohemagglutinin-P (Sigma Chemical Co. St. Louis, MO).

For viability testing, dilutions were made of tenofovir and vehicle control gels in the appropriate cell culture medium to test for cell viability. Dilutions used included 1∶5 (2 mg/ml), 1∶10 (1 mg/ml), 1∶20 (500 µg/ml), 1∶25 (400 µg/ml), 1∶40 (250 µg/ml), 1∶50 (200 µg/ml), and 1∶100 (100 µg/ml). For cell viability, Caco-2 or HEC-1-A cell lines or PBMCs were plated in triplicate in a 96-well plate for each treatment. Diluted tenofovir and vehicle control gels were added to the appropriate wells. Control wells with no treatment (cells only) and medium only were included for background luminescence. The plate was cultured for 24 h and then was washed twice with Hank's Balance Salt Solution (Ca++/Mg++-free) (HBSS). After the last wash, CellTiter-Glo™ (Promega Corp., Madison, WI) was added to all the wells per the manufacturer's instructions and luminescence was measured using a Beckman DTX 880 plate reader. Viability was determined based on deviations from the cell only control and presented as % viability of control ± standard deviation.

Additional 5 day viability testing was done with the epithelial cell lines using the nontoxic concentrations of tenofovir and vehicle control gels. Appropriate wells were exposed for 2 h a day for 5 days. Control wells with no treatment (cells only) and medium only were included for background luminescence. On day 5, the wells were washed and viability was measured as described [28].

To determine the effect of tenofovir and vehicle control gels on epithelial integrity, the transepithelial resistance (TER) was measured as described previously [28]. HEC-1-A or Caco-2 cells were grown on transwell supports until confluency was reached and a polarized monolayer developed as measured by a MilliCell-ERS resistance system (Millipore, Billerica, MA). At that time, a nontoxic concentration (1∶10 or 1 mg/ml) of products were added to the apical surface of the monolayer and resistance readings were measured as indicated. As controls, wells with cells alone, cells treated with N9, or no cells were used. The epithelial resistance was expressed as (Ω x cm^2^) of the treated wells - (Ω x cm^2^) of the no cell wells.

#### Explant culture testing

Normal human ectocervical and colorectal tissues were used. Polarized explant cultures were set-up as previously described [29], [30]. Briefly, the explant was placed with the luminal side up in a transwell. The edges around the explant were sealed with Matrigel™ (BD Biosciences, San Jose, CA). The explants were maintained with the luminal surface at the air-liquid interface. The lamina propria was immersed in medium for ectocervical explants or resting on medium-soaked gelfoam for colorectal explants. Cultures were maintained at 37°C in a 5% CO_2_ atmosphere.

The explants were prepared on day of surgery in duplicate. To ensure even spread of the gels and to allow it to be mixed with HIV-1 for the efficacy testing (below), a 1∶5 dilution of tenofovir or vehicle control gels was applied to the apical side of the explants for 18 h. As controls, explants were untreated or a 1∶5 dilution of 3% N9 gel was applied apically. The next day, explants were washed and viability was evaluated using the MTT [1-(4,5-dimethylthiazol-2-yl)-3,5-diphenylformazan] assay and histology [29], [30].

### Efficacy Testing

To determine ability to prevent HIV-1 infection, PBMCs and tissue explant cultures were treated apically with tenofovir gel. To simulate systemic dosing, 1 mg/ml of tenofovir was added to the basolateral side of the tissue explant cultures.

#### PBMC testing

PBMCs (10^6^ cells/well) were incubated in medium containing HIV-1 (100 TCID_50_/well) and a 1∶20 dilution (0.5 mg/ml) of tenofovir or vehicle control gels, or medium alone (control) for 4 h. The PBMCs were washed 3× with HBSS and cultured in medium at 10^6^ cells/ml/well in triplicate in 48-well plates for 14 days. Culture supernatants were collected every 3 to 4 days and replaced with fresh medium. The supernatants were stored at −80°C until they were assayed for HIV-1 using an HIV-1 p24gag protein ELISA (Perkin Elmer Life and Analytical Sciences, Inc., Waltham, MA). The isolates used included HIV-1_BaL_ purchased from Advanced Biotechnologies, Inc. (Columbia, MD), and primary isolates [HIV-1_C959_ (CCR5), HIV-1_C012_ (CCR5), and HIV-1_A103_ (CXCR4/CCR5)] obtained from the NIH AIDS Research and Reference Reagent Program, Division of AIDS, NIAID, NIH.

#### Explant culture testing

The explants were assembled as described above. To determine the effect of tenofovir gel on HIV-1 infection, a 1∶5 dilution of tenofovir or vehicle control gel was mixed with HIV-1_BaL_ and added to the apical side of the explants. For the systemic application, 1 mg/ml of tenofovir was added to the basolateral compartment and HIV-1_BaL_ was added 15 min later to the apical side of the explants. Eighteen hours after either application the explants were washed and fresh medium (without drug) was added to the basolateral compartment. Every 3 to 4 days over a 3 week period, supernatant was collected and stored at -80°C for HIV-1 p24gag analysis and fresh medium (without drug) was replenished. Immunohistochemistry (IHC) was performed only on ectocervical tissue for HIV-1 infected cells by staining for p24gag [30].

To determine if either formulated or unformulated tenofovir could prevent post-HIV-1 infection, HIV-1_BaL_ was added to the apical side of the ectocervical or colorectal explant cultures. Fifteen or 60 min later, a 1∶5 dilution of tenofovir gel (or vehicle control gel) was added to the apical side of the explants or tenofovir (1 mg/ml) in medium was added to the basolateral side of the explants and allowed to culture for an additional 17 h. The tissues were washed and followed as described above.

### Statistical Analysis

All statistical analyses were performed using SAS version 9.1.3 on SunOS 5.9 platform.

To model the relationship between the *in vitro* released amount of drug and the square root of release time, the lack-of-fit test, using the F statistic, was used to assess the linear relationship versus quadratic relationship. Because the linear relationship provides a better fit to the data, a simple linear regression model was used to estimate the drug release rate.

For tenofovir gel safety testing, epithelial cell lines, PBMCs, and explant tissue treated with tenofovir gel or vehicle control gel were compared to controls (no treatment group) for viability using the one-sided nonparametric Wilcoxon Sign Rank test.

The epithelial monolayer integrity (TER) between diluted tenofovir (1∶10), vehicle control (1∶10), N9 (1∶150), and controls was compared using a one-way analysis of variance. Multiple comparisons between various drug groups versus control were adjusted using the Dunnett's procedure.

To evaluate the efficacy of tenofovir gel in PBMCs against HIV-1 subtypes, comparisons of log_10_ transformed p24gag levels with vehicle control gel or controls (HIV-1 only) were performed at the peak replication time-points. To evaluate the efficacy of tenofovir gel, vehicle control gel, or tenofovir in ectocervical and colorectal explant cultures, comparisons of log_10_ transformed p24gag levels with controls (HIV-1 only) were performed at time-points with peak median p24 gag level for control (HIV-1 only) group. One-sided nonparametric Wilcoxon sign rank test was conducted to assess the efficacy difference.

## Results

### Tenofovir Gel Formulation Evaluation

It is required that formulations be tested to predict how the product will function when applied to the mucosal surface. The major physicochemical properties typically evaluated for semi-solids include osmolality, pH, viscosity, and *in vitro* release. Osmolality is measured to understand how the drug product deviates from normal isosmolar (290 mmol/kg) conditions. This is important because hyperosmolar products can cause mucosal tissue damage [31]. Tenofovir gel and its vehicle control gel had osmolality values which were 11.5-fold (3347 mmol/kg) and 11-fold (3189 mmol/kg), respectively, greater than isosmolar conditions. Both gels were formulated at pH 4.4 which is similar to the vaginal environment. The pH was re-tested after a 1∶5 dilution in cell culture medium and both gels had a pH of 6.4

The viscosities of the tenofovir and vehicle control gels measured at a shear rate of 30 revolutions per minute (rpm) were 2736 centipoise (cps) and 2879 cps, respectively. These results were reproducible in duplicate trials. Both gels were shear thinning in nature, in that the viscosities decreased as applied shear stress increased. The viscosities of tenofovir and vehicle control gels were comparable to over-the-counter lubricant products KY Jelly (2765 cps) and Astroglide Gel (2071 cps).

**Figure 2:**
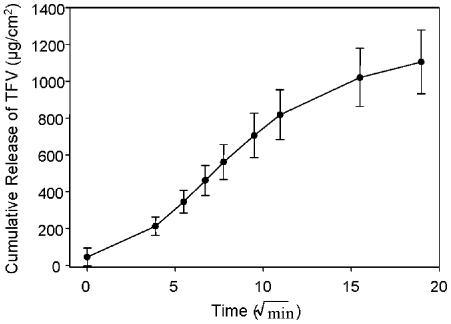
Tenofovir releases from the formulated gel. The *in vitr*o release data show the release profile of tenofovir from the gel; the slope of the line represents the release rate of the product. The data shown represent the mean ± standard deviation of 13 replicates.

For efficacy to be achieved from a dosage form, it is imperative that the drug be released from the product in an efficient and reproducible manner. The *in vitro* release rate reflects how the semi-solid formulation functionally delivers tenofovir from the dosage form. *In vitro* release studies were conducted to determine the release profile of tenofovir from the gel product. In these studies a fairly linear and reproducible release profile was obtained for the gel product (Figure 2). The release rate of tenofovir from the gel product formulation used in these studies was found to be 66.65 µg/cm^2^/min^1/2^.

**Figure 3:**
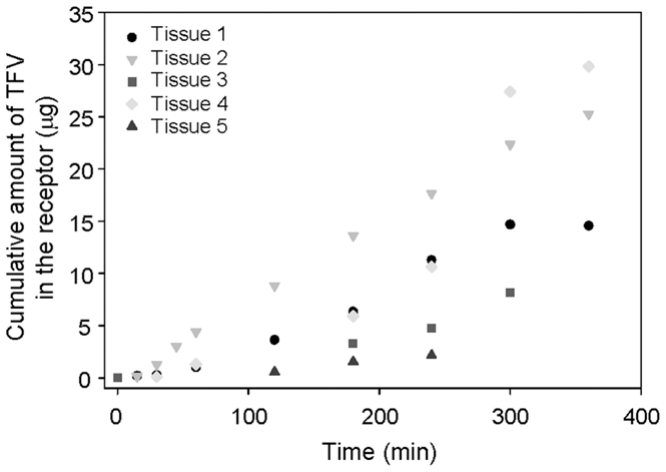
Tenofovir permeates through ectocervical tissue. Tissue permeability data is shown as the cumulative amount of tenofovir which permeated through excised human ectocervical tissue into the receptor chamber of a Franz cell over time. The permeability data represent five separate tissue donors. Tissue permeability data illustrate the intra- and inter-patient variability.

The potential for systemic uptake of tenofovir when delivered topically is assessed through permeability studies. In these studies, the potential for diffusion of tenofovir across an excised human tissue barrier was evaluated in a Franz cell model using ectocervical tissue obtained from separate individuals. Both inter- and intra-patient variability was observed between tenofovir diffusion profiles obtained from different ectocervical samples (Figure 3). Regardless, in all experiments, some amount of tenofovir permeated the excised ectocervical tissue as illustrated by the detectable levels of drug in the receptor chamber following exposure to either drug solution or formulated tenofovir gel product for a 30 min period. However, the amount of tenofovir present in the receptor chamber at later time points differed among separate ectocervical samples tested. These data indicate a potential for variability in the absorption and permeation of tenofovir following topical administration between individual ectocervical tissues.

### Safety of Tenofovir Gel

#### Effect of tenofovir gel on vaginal flora

Normal vaginal flora consists of predominately *Lactobacillus* species; four different *Lactobacillus* species were tested for growth inhibition and toxicity. Other bacteria associated with bacterial vaginosis (*Gardnerella vaginalis* and two *Prevotella* species) and *Neisseria gonorrhoeae* were also tested. Tenofovir (1 mg/ml) was not toxic and did not inhibit the growth of any of the eight bacterial species tested. The gel formulation was tested against the same strains of *L. cripatus*, *L. jenseni*, *L. vaginalis*, and *G. vaginalis* and there was no loss of viability.

#### Effect of tenofovir gel on viability of epithelial cell lines and PBMCs

**Figure 4:**
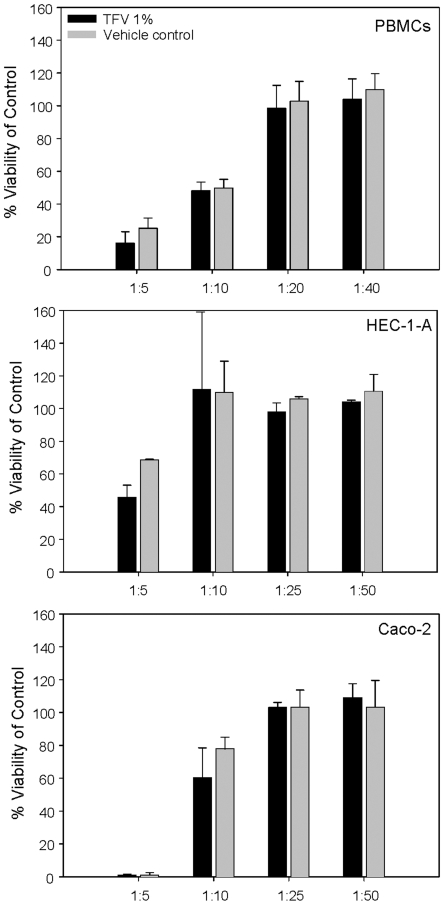
Viability of peripheral blood mononuclear cells (PBMCs) and epithelial cell lines after culture with tenofovir gel. PBMCs or epithelial cell lines (HEC-1-A or Caco-2) were cultured for 24 hours in dilutions of tenofovir or vehicle control gel. Dilutions were made in the appropriate medium for each cell type. Cell viability was measured using CellTiter-Glo® according to the manufacturer's instructions and was calculated as described in the Methods section. The dilution that was used for subsequent work was the lowest dilution to result in ≥60% viability of the cells. The data shown represent the mean ± standard deviation of 5 independent experiments performed in triplicate.

Dilutions of the tenofovir and vehicle control gels were cultured with the epithelial cells and PBMCs for 24 hours and viability was determined as compared to the untreated control. At a dilution of 1∶5, the tenofovir gel showed toxicity (<60% viability) to the HEC-1-A and Caco-2 epithelial cell lines. At a dilution of up to 1∶10, the tenofovir gel showed toxicity (<60% viability) to the PBMCs. Subsequent dilutions resolved the toxicity. Therefore, the minimum nontoxic dilution of the tenofovir gel was 1∶10 (1 mg/ml) for the epithelial cell lines and 1∶20 (0.5 mg/ml) for the PBMCs. The vehicle control gel showed similar results to the epithelial cell lines and PBMCs when diluted 1∶10 or 1∶20 beyond their original formulations (Figure 4).

When these nontoxic dilutions were added for 2 h per day for 5 days to the epithelial cell lines and viability was assessed, minimal impact on epithelial cell viability was noted. By day 5, the Caco-2 cells retained their viability when cultured with the tenofovir (73±16%) and vehicle control (82±10%) gels. The HEC-1-A cell line showed better viability when cultured with tenofovir (118±15%) and vehicle control (119±23%) gels.

**Figure 5:**
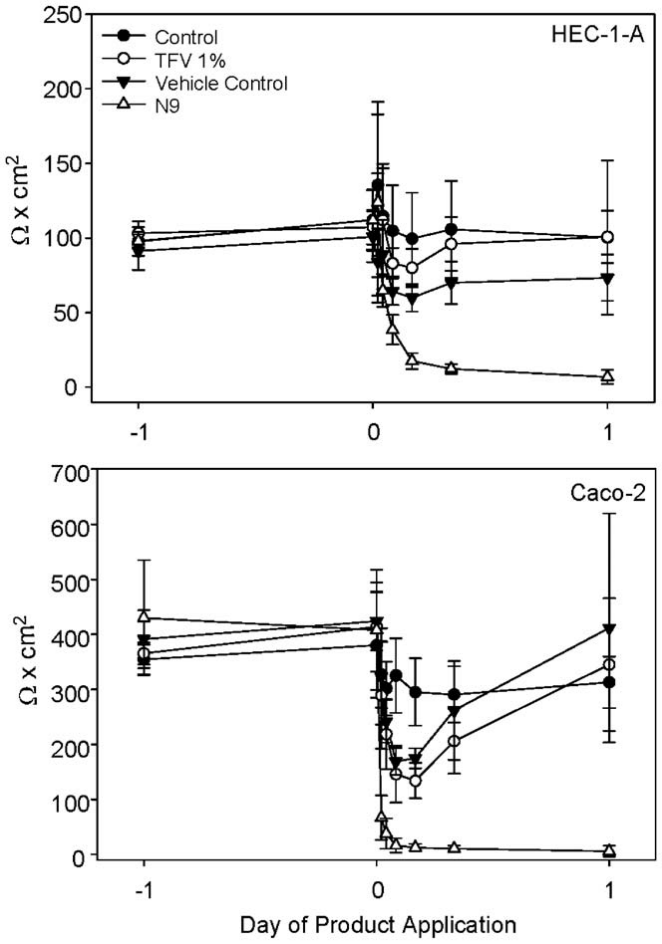
Tenofovir and vehicle control gels transiently reduced epithelial monolayer integrity. HEC-1-A (upper panel) and Caco-2 (lower panel) cells were grown in transwell supports until they formed stable monolayers. A 1:10 dilution of tenofovir or vehicle control gels were added to the apical chamber at t = 0 and resistance readings were measured at 30 min, 1, 2, 4, 8, and 24 h. As a toxicity control, a 1:50 dilution (0.6 mg/ml) of nonoxynol-9 (N9) gel was added to the indicated apical chambers and resistance readings were measured. The data shown represent the mean ± standard deviation of 3 independent experiments performed in duplicate.

For testing the epithelial monolayer integrity, 1∶10 dilutions of tenofovir and vehicle control gels were added to monolayers of HEC-1-A and Caco-2 cells and the impact on the monolayer was measured over 24 h. As a toxicity control, a 1∶50 dilution of a 3% N9 gel was added to separate wells. Control wells (cells treated with medium only) for HEC-1-A (upper panel) and Caco-2 (lower panel) cells varied by no more than 24% in their TER over the course of the 24 h period (Figure 5). The diluted tenofovir gel reduced the TER of the HEC-1-A monolayer by 9% (not significant) and the Caco-2 monolayer by 55% (*p* = .0026) at 4 h after application as compared to the control wells. Similar results were found using the diluted vehicle control gel. However, with both cell lines, the TER returned to control levels by 24 h after either product application indicating the disruption of the TER was transient. Conversely, N9-treated cell monolayers continued to lose their TER with total loss occurring by 4 h after application.

#### Effect of tenofovir gel on viability of ectocervical and colorectal explants

**Figure 6:**
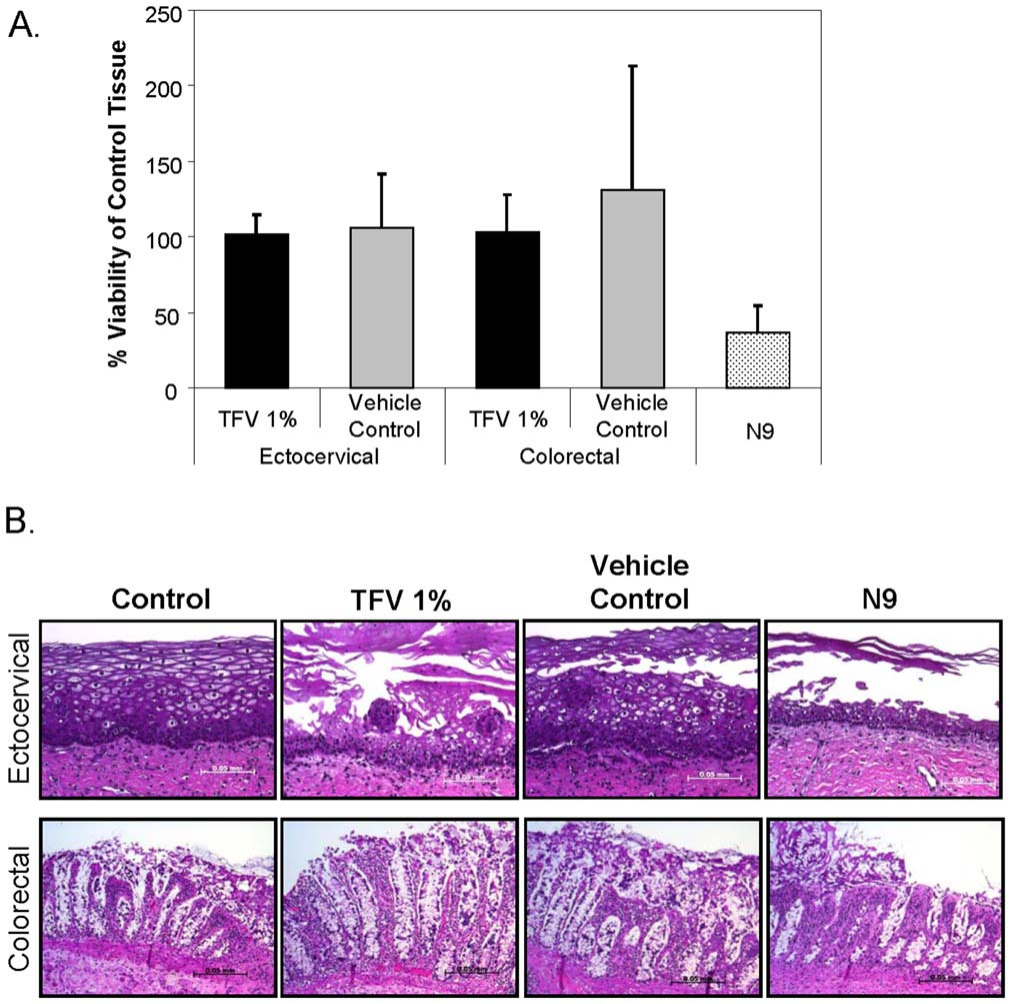
Viability of explant cultures after a 24 hour exposure to tenofovir or vehicle control gels. Ectocervical and colorectal explants were polarized and tenofovir or vehicle control gels were diluted 1:5 in the appropriate culture medium and applied to the apical surface. A 1:5 dilution of nonoxynol-9 (N9) was applied apically to the explants at the same time as the tenofovir and vehicle control gels as a toxicity control. Untreated explants were the negative control tissues. The explants were cultured for 24 h, washed five times, and placed in either medium containing 1-(4,5-dimethylthiazol-2-yl)-3,5-diphenylformazan (MTT) to assess tissue viability by measuring mitochondrial activity (A) or formalin to fix the tissue for hematoxylin-eosin staining for histology (original magnification 20×; bar length 0.05 mm) (B). Explants from five tissue donors were evaluated for viability after exposure to topical gels. The % viability was determined by dividing the corrected optical density of the treated explant by the corrected optical density of the control explant. Histology shown is representative of the five tissues evaluated.

To assess tenofovir and vehicle control gels impact on tissue viability, ectocervical and colorectal explant cultures were set-up polarized, in transwells on the day of surgery. A 1∶5 dilution, to ensure even spread over the tissue, of each product was made in the appropriate medium. The diluted gels were applied to the epithelial surface of the tissue and remained in contact for 24 h. The tissues were washed and prepared for the MTT assay or histology. While no decrease in viability was noted when using the MTT assay (Figure 6A), which measures mitochondrial activity, epithelial fracture was noted in 3 of 5 ectocervical and colorectal tissues cultured with tenofovir or vehicle control gels (Figure 6B). The lamina propria appeared intact. As a control, N9 gel was prepared in a similar fashion and applied to the tissues and showed a 60% reduction in viability (*p* = .03) as determined by the MTT assay and stripping of the epithelium from the lamina propria by histology in all tissues tested (Figure 6B).

### Efficacy of Tenofovir

#### Efficacy testing of tenofovir gel in PBMCs

**Figure 7:**
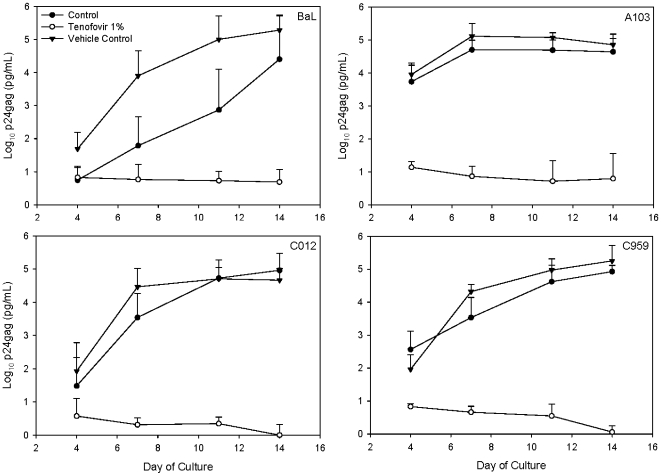
Efficacy of tenofovir against primary isolates of HIV-1. Peripheral blood mononuclear cells were activated and cultured with HIV-1 (BaL, laboratory-adapted CCR5-using clade B isolate; A103, primary CCR5/CXCR4-using clade A isolate; C012, primary CCR5-using clade C isolate; and C959, primary CCR5-using clade C isolate) with or without tenofovir or vehicle control gel. After 4 hours, the cultures were washed and fresh medium was added. Supernatant was collected every 3 to 4 days and stored at -80°C. HIV-1 infection was followed using a p24gag ELISA. The data shown represent the log_10_-transformed (pg/mL) ±95% confidence interval of 4 (BaL) or 5 (A103, C012, and C959) independent experiments.

Tenofovir is currently being used as a therapeutic (TDF; Viread®) and has shown extensive activity against numerous HIV-1 subtypes [16]. However, the formulated tenofovir gel has not been formally tested for efficacy against HIV-1 subtypes. To fill this gap, the 1∶20 dilution of tenofovir or vehicle control gel was mixed with HIV-1_BaL_ or one of three primary HIV-1 isolates representing two prominent subtypes in Africa (subtypes A and C) and cultured with PBMCs. Tenofovir gel reduced HIV-1 infection from 2.7 to 4.7 log_10_ (*p* = .03) as compared to the peak p24 levels of the control cultures (Figure 7). A similar reduction in HIV-1 growth was noted between tenofovir and vehicle control gel treated cultures. Tenofovir gel was effective at blocking HIV-1 infection regardless of subtype.

#### Efficacy testing of tenofovir gel and tenofovir in explant cultures

**Figure 8:**
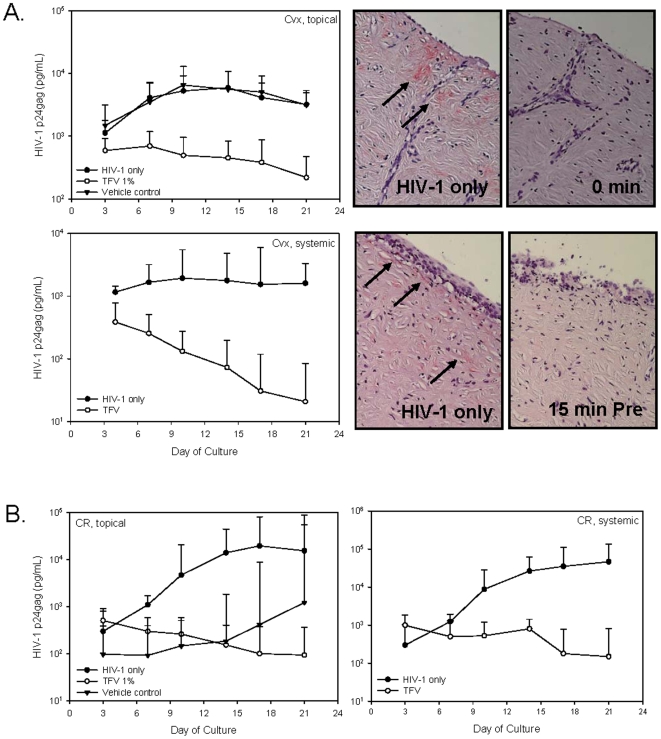
Tenofovir applied topically or systemically protect ectocervical and colorectal explants from HIV-1 infection. Tenofovir or vehicle control gels were mixed with HIV-1_BaL_ and applied to the apical side of ectocervical (A) or colorectal (B) explants. In a separate study to simulate systemic dosing, tenofovir (1 mg/ml) was added to the basolateral side of the explants 15 min prior to the addition of HIV-1_BaL_ to the apical side of the tissue. After an overnight incubation, the explants were washed and cultured for 21 days with medium sampled and replaced every 3 to 4 days. HIV-1 replication was monitored in the supernatants using a p24gag ELISA. The data shown represent the median ±95% confidence interval of a minimum of 3 independent tissues performed in duplicate. The wide confidence intervals reflect the p24 variability between tissue donors. For ectocervical tissue, immunohistochemistry for p24gag positive cells of the day 21 explants was done and representative pictures are shown. Arrows indicate p24gag positive cells.

Tenofovir could be used in either a topical or systemically available dosage form for prevention studies. To determine whether topical and systemic dosing could be modeled in explant cultures and if both are effective against HIV-1, ectocervical and colorectal explant cultures were set-up as described [29], [30]. For topical administration, HIV-1 was mixed with tenofovir or vehicle control gel and added to the apical side of the cultures for overnight incubation. To simulate systemic administration, 1 mg/ml of tenofovir was added to the basolateral supernatant of the cultures for 15 min and then HIV-1 was applied to the apical side of the culture for overnight incubation. Tenofovir, whether applied apically or basolaterally prevented HIV-1 infection of ectocervical and colorectal explant cultures. HIV-1 p24gag was reduced by 1.1 log_10_ (day 10) in topically-treated and 1.6 log_10_ (day 10) in basolaterally-treated ectocervical explants as compared to untreated (HIV-1 only) or vehicle control-treated explants. This protection was confirmed by IHC for HIV-1 p24gag positive cells in the untreated (Figure 8A; upper and lower right panels) and vehicle control-treated tissues (data not shown) with no detectable IHC positive cells in the tenofovir-treated tissue. In colorectal tissues (Figure 8B), not only did tenofovir delivered either apically or basolaterally reduce HIV-1 p24gag production, by 2.1 log_10_ (day 18) and 2.0 log_10_ (day 18) respectively, but the vehicle control gel reduced HIV-1 p24gag production by 1.3 log_10_ (day 21). Despite the reduced p24gag, the levels increased over time indicating that the explants were not protected by the vehicle control gel.

**Figure 9:**
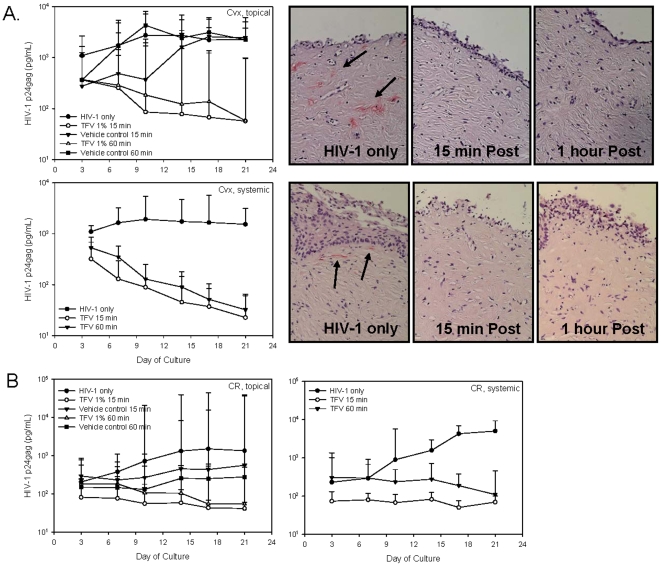
Tenofovir protects ectocervical and colorectal explants up to 60 minutes after HIV-1 exposure. Ectocervical (A) and colorectal (B) explants were apically treated with HIV-1_BaL_; 15 min or 60 min later tenofovir or vehicle control gels were applied to the apical surface or tenofovir (1 mg/ml) was added to the basolateral side of the explants. After incubating overnight, the explants were washed and cultured for 21 days with medium sampled and replaced every 3 to 4 days. HIV-1 replication was monitored in the supernatants using a p24gag ELISA. The data shown represent the median ±95% confidence interval of a minimum of 3 independent tissues performed in duplicate. The wide confidence intervals reflect the p24 variability between tissue donors. For ectocervical tissue, immunohistochemistry for p24gag positive cells of the day 21 explants was done and representative pictures are shown. Arrows indicate p24gag positive cells.

With the success of the topical and systemic applications applied concurrently or just before the addition of HIV-1, we wanted to know whether delayed application or post-exposure prophylaxis of drug could prevent explant infection. Tenofovir gel (or vehicle control gel) (apical) or tenofovir (basolateral) was added to the appropriate culture either 15 min or 60 min after the addition of HIV-1 to the explants. The delayed addition of tenofovir protected the ectocervical (Figure 9A) and colorectal (Figure 9B) explants as shown by similar log_10_ reductions of HIV-1 p24gag protein to those noted above and no detectable infected cells at study endpoint (immunohistochemistry; ectocervical tissue only). The vehicle control gel did not reduce HIV-1 infection of the ectocervical explants, but did delay or reduce HIV-1 infection in the colorectal explants similar to what was observed for concurrent administration of gel and virus above.

## Discussion

Prevention efforts remain a high priority in the approach to control the global HIV-1 epidemic. The current microbicides being developed are specific for HIV-1 and may be more potent than the first generation, non-specific microbicides. These next generation microbicides utilize effective therapeutics or specific anti-HIV-1 targets. Tenofovir gel is the lead candidate being brought forward in efficacy clinical trials. Also, interest is increasing in using oral PrEP for HIV-1 prevention. We have utilized our testing algorithm to evaluate tenofovir gel and the unformulated drug, tenofovir, for safety and efficacy against HIV-1. The tenofovir gel has an acidic pH, is shear thinning, and is hyperosmolar. Tenofovir is reproducibly released from the gel and is able to permeate into the tissue resulting in the prevention of HIV-1 infection of the ectocervical and colorectal explant tissues. When used to simulate systemic dosing (basolateral application), the drug is effective in preventing HIV-1 infection of the ectocervical and colorectal tissues. Collectively, our findings suggest that tenofovir, whether used topically (vaginally or rectally) or systemically, will be effective against HIV-1 infection.

As the development of topical microbicides advances, the importance of formulation is becoming better appreciated. General desirable aspects of formulations include safety (e.g. isosmolar aqueous gels), efficacy, stability, and patient acceptability. More specific formulation aspects include optimal retention time, appropriate drug diffusion, and targeted drug delivery. The *in vitro* release data allows us to infer the amount of drug released by the product. The cumulative release of approximately 670 µM of tenofovir from the tenofovir gel occurred over the six hours of the assay. By 15 minutes, 134 µM of tenofovir is released from the gel which is 67 times greater than the 2 µM IC_50_ needed to block HIV-1 replication *in vitro*. Four of five tissues studied had permeability levels above the 2 µM IC_50_ by three hours and the least permeable tissue achieved this level within 4 hours. The variability observed among these tissues is consistent with the variable amounts of drug detected in the blood of women in a clinical trial [19]. Importantly, detectable levels of tenofovir in the blood (an indication of permeation through the tissue) have been an indicator of protection in a non-human primate model [17]. The permeability data presented here predict the amount of drug which will be systemically available upon exposure. However the evaluation of tenofovir levels in the tissue is also important because of local HIV-1 target cells. For this reason we are currently determining the amount of tenofovir that permeates into the ectocervical and colorectal explants and will correlate these levels to protection against HIV-1 infection.

One of the important gaps in the field of microbicide research is the ability to link pre-clinical safety and efficacy testing to clinical outcomes for validation of these assays. Frequently products will show efficacy against HIV-1 *in vitro*, but result in no benefit or possibly increased HIV-1 infection during a clinical trial as was the case for N9 and cellulose sulfate. Because N9 was already available as an over-the-counter spermicide and early work indicated that it killed HIV-1 and *Neisseria gonorrhoeae* [32], [33], N9 was clinically tested as a microbicide. Early clinical trials indicated it was acceptable and safe [34], [35], [36], [37]. However, two of three efficacy trials were halted early due to evidence of harm, i.e. increased risk of HIV-1 infection [38], [39], [40]. Subsequent studies, including our own, showed extensive toxicity and damage to epithelial cells and ectocervical and colorectal explants [28], [29], [30], [41], [42], [43], [44]. Our bacteriological studies determined most *Neisseria gonorrhoeae* isolates were resistant to N9 which explains why the women were not protected from infection [24]. The use of N9 in our pre-clinical algorithms is now relegated to the toxicity control. While we have not tested cellulose sulfate using our pre-clinical algorithm, we have tested formulated 4% and 0.5% PRO 2000 and Carraguard gels. We showed that the 4% PRO 2000 gel reduced the transepithelial resistance in epithelial cells [28] and demonstrated toxicity in ectocervical and colorectal explant tissues [29], [30]. The 0.5% PRO 2000 and vehicle control gels showed no such toxicity. Moreover, the 0.5% PRO 2000 gel was effective at preventing HIV-1 infection of PBMCs and explant tissues [28], [29], [30]. The 2% PRO 2000 arm, but not the 0.5% arm, was halted due to futility in the MDP301 trial evaluating these PRO 2000 gels for the prevention of vaginally acquired HIV-1 infection. The HPTN 035 trial showed that 0.5% PRO 2000 reduced HIV-1 infection by 30% as compared to the placebo or condom only arms [11]. Unfortunately, this result for 0.5% PRO 2000/5 gel was not statistically significant, and was confirmed by the recent release of results from the MDP301 study [15]. These data are consistent with the PRO 2000 findings in our pre-clinical algorithm and others [45]. Even though PRO 2000 appeared effective in *in vitro* experiments, semen reduced its efficacy in animal model testing which may explain the lack of protection in the clinical trials. While not presented here, we have tested the efficacy of tenofovir in the presence of semen and have found no reduction of activity against HIV-1 [46]. Further, we showed that while Carraguard was quite safe, it was not effective in preventing HIV-1 infection of PBMCs (especially with primary HIV-1 subtypes), cell-to-cell transmission of HIV-1, or HIV-1 infection of ectocervical explant cultures [28], [30]. These data are consistent with the clinical trial findings showing that Carraguard was safe and acceptable to the women using it, but did not prevent HIV-1 infection [14]. The parallel results of our pre-clinical testing of formulated products and those of the clinical trials suggest our algorithm should be predictive of clinical outcomes.

Our work with tenofovir gel shows a transient reduction in the transepithelial resistance of the epithelial cells and fracture of the ectocervical and colorectal epithelium. Because similar observations were made with the vehicle control gel, these effects can be attributed to the hyperosmolar nature of the formulation. These subtle *in vitro* changes are in contrast to the results from clinical trials which did not show any safety concerns as determined by self reports or colposcopic measurements [19], [20]. It should be noted that the placebo used for this study was the vehicle control and not the Universal Placebo (hydroxyethylcellulose; [47]). Because the Universal Placebo is isosmolar, its use in clinical trials could show potential safety concerns of the active gels. The discrepancies between our findings and those of the clinical studies may be due to 1) the length of time of exposure to the gel, 2) the dilution of gel used for the pre-clinical testing, 3) not having a “no product” or true placebo arm to control for potential genital changes from background in these populations, 4) subjectivity of self reporting, 5) timing of sample collection to capture attributable changes, and/or 6) sensitivity of colposcopic testing to detect the subtle, transient changes in the epithelium. For points 1 and 2, there is little published data regarding how long a gel is retained in the vagina or rectum and how mucosal fluids dilute the gel *in situ*. We utilized a 1∶5 dilution of the gels for testing, because these gels are viscous and hard to manipulate for culture conditions. For point 3, the use of the Universal Placebo gel may obviate the need for a “no product” arm. It is important to note for points 4 and 5, daily use of vaginally or rectally applied N9 by volunteers was well tolerated [37], [48], [49] and for point 6, the timing of when colposcopy or other testing is done in relationship to the last product dose was important [44], [50]. A recent colposcopy study showed that there was no increase in genital lesions in women using various N9 formulations compared to condom only users [51]. However, N9 was used coitally and colposcopy was not standardized after product use. Phillips et al. [44], [50] evaluated a N9-containing gel applied rectally by taking rectal lavages and biopsies at 15 min through 12 h post dose. Denudation of the epithelium was found at 15 min with completely normal epithelium by 12 h. These data illustrate that variability can be found during clinical studies and interpretation of the data is dependent on when samples are taken. While explant testing performed for this study regarding N9 toxicity findings was consistent with previous studies [29], [30], explant cultures have limitations that include 1) lack of hormone modulation (ectocervical tissue), 2) no recruitment of immune cells, and 3) inability to regenerate/repair. While epithelial fracture was observed in the majority of ectocervical and colorectal explants after tenofovir gel application, no damage was observed in the lamina propria. The inability of the explants to repair makes this system more sensitive to potential changes by topical formulations than *in vivo* use.

Unformulated tenofovir has previously been evaluated in rectal explants for efficacy [52], [53]. For this system, tissue pieces were placed unpolarized into tissue culture wells with varying concentrations of tenofovir and a standard amount of HIV-1. Under these conditions, tenofovir was shown to be effective against HIV-1 infection. However, these data are hard to extrapolate to the gel that will be used in clinical trails. Using our more stringent model of polarizing the tissue, topical application of formulated tenofovir gel was protective against HIV-1. Further, our polarized model also allows us to simulate systemic dosing of drug by applying tenofovir (1 mg/ml) to the basolateral side of the tissue (lamina propria) and HIV-1 to the apical side of the tissue (epithelium). Using this system, tenofovir applied basolaterally was effective at preventing HIV-1 infection of the explants. Application of tenofovir gel apically or tenofovir basolaterally 15 to 60 minutes after HIV-1 challenge was also protective. The efficacy of tenofovir when used clinically is still not known, but the data provided here suggest that tenofovir will be effective topically or systemically to prevent the sexual transmission of HIV-1.

Pre-clinical testing of microbicide products has greatly advanced this past decade. Innovative systems are being used and developed to recapitulate human exposure as best we can. While we still do not know the intricacies of how HIV-1 is transmitted, comprehensive pre-clinical algorithms such as those described here provide important basic science information while supplementing and guiding animal and human studies. Vaginal and rectal exposure models in non-human primates have shown the feasibility of tenofovir as a topical microbicide and oral PrEP by protecting the majority of the animals challenged with SHIV [17], [18], [54]. The inclusion of biopsies from early clinical studies to challenge with HIV-1 *ex vivo* may allow us to more accurately determine the efficacy of the drug in subsequent clinical trials. This concept is supported by a recent study using a non-nucleoside reverse transcriptase inhibitor, UC781, applied rectally with biopsies taken at defined time points after gel application. The biopsies taken 30 minutes after gel application were protected from HIV-1 infection [55]. The data presented here suggest that a thorough pre-clinical evaluation of the formulated microbicide is needed as effective candidates are identified and before they move into clinical trials.

## References

[1] UNAIDS/WHO (2008). Report on global AIDS epidemic..

[2] Gray RH, Wawer MJ, Polis CB, Kigozi G, Serwadda D (2008). Male Circumcision and Prevention of HIV and Sexually Transmitted Infections.. Curr Infect Dis Rep.

[3] Mills E, Cooper C, Anema A, Guyatt G (2008). Male circumcision for the prevention of heterosexually acquired HIV infection: a meta-analysis of randomized trials involving 11,050 men.. HIV Med.

[4] Steinbrook R (2007). One step forward, two steps back–will there ever be an AIDS vaccine?. N Engl J Med.

[5] Celum C, Wald A, Hughes J, Sanchez J, Reid S (2008). Effect of aciclovir on HIV-1 acquisition in herpes simplex virus 2 seropositive women and men who have sex with men: a randomised, double-blind, placebo-controlled trial.. Lancet.

[6] Greig FE, Koopman C (2003). Multilevel analysis of women's empowerment and HIV prevention: quantitative survey Results from a preliminary study in Botswana.. AIDS Behav.

[7] Kalichman SC, Williams EA, Cherry C, Belcher L, Nachimson D (1998). Sexual coercion, domestic violence, and negotiating condom use among low-income African American women.. J Womens Health.

[8] Carballo-Dieguez A, Miner M, Dolezal C, Rosser BR, Jacoby S (2006). Sexual negotiation, HIV-status disclosure, and sexual risk behavior among Latino men who use the internet to seek sex with other men.. Arch Sex Behav.

[9] Olmsted SS, Khanna KV, Ng EM, Whitten ST, Johnson ON (2005). Low pH immobilizes and kills human leukocytes and prevents transmission of cell-associated HIV in a mouse model.. BMC Infect Dis.

[10] Tuyama AC, Cheshenko N, Carlucci MJ, Li JH, Goldberg CL (2006). ACIDFORM inactivates herpes simplex virus and prevents genital herpes in a mouse model: optimal candidate for microbicide combinations.. J Infect Dis.

[11] Abdool Karim S, Coletti A, Richardson B, Ramjee G, Hoffman I Safety and effectiveness of vaginal microbicides BufferGel and 0.5% PRO 2000/5 gel for the prevention of HIV infection in women: results from the HPTN 035 trial. CROI 2009; Montreal, Canada..

[12] Moszynski P (2007). Halt to microbicide trial sets back AIDS research.. BMJ.

[13] Van Damme L, Govinden R, Mirembe FM, Guedou F, Solomon S (2008). Lack of effectiveness of cellulose sulfate gel for the prevention of vaginal HIV transmission.. N Engl J Med.

[14] Skoler-Karpoff S, Ramjee G, Ahmed K, Altini L, Plagianos MG (2008). Efficacy of Carraguard for prevention of HIV infection in women in South Africa: a randomised, double-blind, placebo-controlled trial.. Lancet.

[15] Microbicide Development Programme (2009). HIV ‘prevention’ gel PRO 2000 proven ineffective.. http://www.mdp.mrc.ac.uk/archive.html.

[16] Palmer S, Margot N, Gilbert H, Shaw N, Buckheit R (2001). Tenofovir, adefovir, and zidovudine susceptibilities of primary human immunodeficiency virus type 1 isolates with non-B subtypes or nucleoside resistance.. AIDS Res Hum Retroviruses.

[17] Cranage M, Sharpe S, Herrera C, Cope A, Dennis M (2008). Prevention of SIV Rectal Transmission and Priming of T Cell Responses in Macaques after Local Pre-exposure Application of Tenofovir Gel.. PLoS Med.

[18] Parikh UM, Dobard C, Sharma S, Cong ME, Jia H (2009). Complete protection from repeated vaginal simian-human immunodeficiency virus exposures in macaques by a topical gel containing tenofovir alone or with emtricitabine.. J Virol.

[19] Mayer KH, Maslankowski LA, Gai F, El-Sadr WM, Justman J (2006). Safety and tolerability of tenofovir vaginal gel in abstinent and sexually active HIV-infected and uninfected women.. AIDS.

[20] Hillier SL. Safety and acceptability of coitally dependant use of 1% tenofovir over six months of use; Microbicides 2008; New Delhi, India..

[21] Cosentino LA, Landers DV, Hillier SL (2003). Detection of Chlamydia trachomatis and Neisseria gonorrhoeae by strand displacement amplification and relevance of the amplification control for use with vaginal swab specimens.. J Clin Microbiol.

[22] Antonio MA, Hawes SE, Hillier SL (1999). The identification of vaginal Lactobacillus species and the demographic and microbiologic characteristics of women colonized by these species.. J Infect Dis.

[23] Moncla BJ, Braham P, Rabe LK, Hillier SL (1991). Rapid presumptive identification of black-pigmented gram-negative anaerobic bacteria by using 4-methylumbelliferone derivatives.. J Clin Microbiol.

[24] Moncla BJ, Hillier SL (2005). Why nonoxynol-9 may have failed to prevent acquisition of Neisseria gonorrhoeae in clinical trials.. Sex Transm Dis.

[25] Moncla BJ, Pryke K, Isaacs CE (2008). Killing of Neisseria gonorrhoeae, Streptococcus agalactiae (group B streptococcus), Haemophilus ducreyi and vaginal Lactobacillus by 3-O-octyl-sn-glycerol.. Antimicrob Agents Chemother.

[26] Fogh J, Wright WC, Loveless JD (1977). Absence of HeLa cell contamination in 169 cell lines derived from human tumors.. J Natl Cancer Inst.

[27] Kuramoto H (1972). Studies of the growth and cytogenetic properties of human endometrial adenocarcinoma in culture and its development into an established line.. Acta Obstet Gynaecol Jpn.

[28] Dezzutti CS, James VN, Ramos A, Sullivan ST, Siddig A (2004). In vitro comparison of topical microbicides for prevention of human immunodeficiency virus type 1 transmission.. Antimicrob Agents Chemother.

[29] Abner SR, Guenthner PC, Guarner J, Hancock KA, Cummins JE (2005). A Human Colorectal Explant Culture to Evaluate Topical Microbicides for the Prevention of HIV Infection.. J Infect Dis.

[30] Cummins JE, Guarner J, Flowers L, Guenthner PC, Bartlett J (2007). Preclinical testing of candidate topical microbicides for anti-human immunodeficiency virus type 1 activity and tissue toxicity in a human cervical explant culture.. Antimicrob Agents Chemother.

[31] Fuchs EJ, Lee LA, Torbenson MS, Parsons TL, Bakshi RP (2007). Hyperosmolar sexual lubricant causes epithelial damage in the distal colon: potential implication for HIV transmission.. J Infect Dis.

[32] Jennings R, Clegg A (1993). The inhibitory effect of spermicidal agents on replication of HSV-2 and HIV-1 in-vitro.. Journal of Antimicrobial Chemotherapy.

[33] Malkovsky M, Newell A, Dalgleish AG (1988). Inactivation of HIV by nonoxynol-9.. Lancet.

[34] Martin HL , Stevens CE, Richardson BA, Rugamba D, Nyange PM (1997). Safety of a nonoxynol-9 vaginal gel in Kenyan prostitutes. A randomized clinical trial.. Sex Transm Dis.

[35] Roddy RE, Chutivongse S, Niruthisard S (1992). The effects of frequent nonoxynol-9 use on the vaginal and cervical mucosa.. Lancet.

[36] Rustomjee R, Abdool Karim Q, Abdool Karim SS, Laga M, Stein Z (1999). Phase 1 trial of nonoxynol-9 film among sex workers in South Africa.. AIDS.

[37] Van Damme L, Chandeying V, Ramjee G, Rees H, Sirivongrangson P (2000). Safety of multiple daily applications of COL-1492, a nonoxynol-9 vaginal gel, among female sex workers. COL-1492 Phase II Study Group.. AIDS.

[38] Kreiss J, Ngugi E, Holmes K, Ndinya-Achola J, Waiyaki P (1992). Efficacy of nonoxynol 9 contraceptive sponge use in preventing heterosexual acquisition of HIV in Nairobi prostitutes.. JAMA.

[39] Roddy RE, Zekeng L, Ryan KA, Tamoufe U, Weir SS (1998). A controlled trial of nonoxynol 9 film to reduce male-to-female transmission of sexually transmitted diseases.. N Engl J Med.

[40] Van Damme L, Ramjee G, Alary M, Vuylsteke B, Chandeying V (2002). Effectiveness of COL-1492, a nonoxynol-9 vaginal gel, on HIV-1 transmission in female sex workers: a randomised controlled trial.. Lancet.

[41] Beer BE, Doncel GF, Krebs FC, Shattock RJ, Fletcher PS (2006). In vitro preclinical testing of nonoxynol-9 as potential anti-human immunodeficiency virus microbicide: a retrospective analysis of results from five laboratories.. Antimicrob Agents Chemother.

[42] Niruthisard S, Roddy RE, Chutivongse S (1991). The effects of frequent nonoxynol-9 use on the vaginal and cervical mucosa.. Sex Transm Dis.

[43] Patton DL, Cosgrove Sweeney YT, Rabe LK, Hillier SL (2002). Rectal applications of nonoxynol-9 cause tissue disruption in a monkey model.. Sex Transm Dis.

[44] Phillips DM, Taylor CL, Zacharopoulos VR, Maguire RA (2000). Nonoxynol-9 causes rapid exfoliation of sheets of rectal epithelium.. Contraception.

[45] Patel S, Hazrati E, Cheshenko N, Galen B, Yang H (2007). Seminal plasma reduces the effectiveness of topical polyanionic microbicides.. J Infect Dis.

[46] Kunjara Na Ayudhya RP, Billitto N, Rooney J, Dezzutti C. Influence of Semen on Efficacy of Tenofovir 1% Gel; Keystone Symposia: Prevention of HIV/AIDS 2009; Keystone, Colorado..

[47] Tien D, Schnaare RL, Kang F, Cohl G, McCormick TJ (2005). In vitro and in vivo characterization of a potential universal placebo designed for use in vaginal microbicide clinical trials.. AIDS Res Hum Retroviruses.

[48] Tabet SR, Surawicz C, Horton S, Paradise M, Coletti AS (1999). Safety and toxicity of nonoxynol-9 gel as a rectal microbicide.. Sex Transm Dis.

[49] Van Damme L, Niruthisard S, Atisook R, Boer K, Dally L (1998). Safety evaluation of nonoxynol-9 gel in women at low risk of HIV infection.. AIDS.

[50] Phillips DM, Sudol KM, Taylor CL, Guichard L, Elsen R (2004). Lubricants containing N-9 may enhance rectal transmission of HIV and other STIs.. Contraception.

[51] Harwood B, Meyn LA, Ballagh SA, Raymond EG, Archer DF (2008). Cervicovaginal colposcopic lesions associated with 5 nonoxynol-9 vaginal spermicide formulations.. Am J Obstet Gynecol.

[52] Fletcher PS, Elliott J, Grivel JC, Margolis L, Anton P (2006). Ex vivo culture of human colorectal tissue for the evaluation of candidate microbicides.. AIDS.

[53] Herrera C, Cranage M, McGowan I, Anton P, Shattock RJ (2009). Reverse transcriptase inhibitors as potential colorectal microbicides.. Antimicrob Agents Chemother.

[54] Garcia-Lerma JG, Otten RA, Qari SH, Jackson E, Cong ME (2008). Prevention of rectal SHIV transmission in macaques by daily or intermittent prophylaxis with emtricitabine and tenofovir.. PLoS Med.

[55] Anton P, Elliott J, Tanner K, Cho D, Johnson E Strong suppression of HIV-1 infection of colorectal explants following in vivo rectal application of UC781 gel: a novel endpoint in a Phase I trial. CROI 2009; Montreal, Canada. Abst 1067.

